# Pharmacoprophylaxis of alcohol dependence: Review and update Part II: Efficacy

**DOI:** 10.4103/0019-5545.31515

**Published:** 2007

**Authors:** Sandeep Grover, Debasish Basu, Gaurav Bhateja

**Affiliations:** Drug De-addiction and Treatment Centre, Department of Psychiatry, Postgraduate Institute of Medical Education and Research, Chandigarh, India

**Keywords:** Alcohol dependence, disulfiram, acamprosate, naltrexone, topiramate, efficacy

## Abstract

Alcohol dependence is a major problem in India. The pharmacological armamentarium for relapse prevention of alcohol has widened with the addition of new drugs. In this article, we review the pharmacology and efficacy of the four most important such drugs: disulfiram, naltrexone, acamprosate and topiramate. The first part of this two-part review series concerns the comparative pharmacology and the second part concerns the efficacy studies. Overall, all four of these drugs have modest but clinically significant usefulness as pharmacoprophylactic agents for relapse prevention or minimization of alcohol dependence. Combinations might be helpful, especially for naltrexone and acamprosate. The issue of supervision and compliance remains important, especially for such drugs as disulfiram and naltrexone. Topiramate is a promising new agent and requires further study. Disulfiram, while very effective in compliant patients, presents challenges in terms of patient selection and side effects. For patients with hepatic impairment, acamprosate is a good choice.

## INTRODUCTION

As mentioned in the part 1 of this two-part review, alcohol dependence is a major problem in India. The pharmacological armamentarium for relapse prevention of alcohol has widened with the addition of new drugs. In this article, we review the efficacy of the four most important such drugs: disulfiram, naltrexone, acamprosate and topiramate. The first part of this two-part review series concerned the comparative pharmacology and this second part concerns the efficacy studies.

In this era of managed care and increasing pressures of accountability, healthcare providers need credible scientific information for decision-making purposes in recommending medications. In the current scientific literature, anything less than randomized controlled trials (RCT) are not accepted as evidence when addressing the question of drug efficacy. In this review we will try to limit our discussion to only randomized controlled trials and metanalysis. There are only occasional head-to-head comparisons between any of the above two drugs. In most of the trials the active drugs are compared with placebo, hence considering any one of them to be superior to the other would be wrong. Further the outcome parameters in various studies are different which further makes drawing any conclusion difficult. In the following section we will review the efficacy of disulfiram, naltrexone, acamprosate and topiramate.

## DISULFIRAM

Although disulfiram is oldest among the three drugs, there are fewer well-designed studies compared to acamprosate and naltrexone. The review of studies of disulfiram shows that there are only occasional studies after 1992. The efficacy of disulfiram has been reviewed in detail by Hughes and Cook[1] and Garbutt *et al*.[2] In the following section we will frequently refer to the conclusion drawn by Hughes and Cook.[1] Hughes and Cook[1] reviewed 24 studies between 1967 to 1995; the sample varied from 16 to 605, there where only 4 single blind studies and only 1 double blind study, 13 studies were randomized and 13 studies used matched controls and the mean duration of follow up was 12.9 months with a range of 2 to 24 months. The authors rightly pointed out the difficulties in assessing the efficacy of disulfiram is hampered by the methodological limitations like patient-treatment matching, the impossibility of double blindness, compliance and use of disulfiram as part of treatment package. The authors listed various patient characteristics which offer potential for matching with disulfiram treatment and predicting good outcome. The characteristics includes older age (over 40 years), high social stability, abstinence prior to treatment, being in early abstinence but in crisis, being abstinent but requiring support to comply with the treatment, high motivation, contact with alcoholic anonymous, being relapse-prone but continuing in treatment, low level of depression, no history of blackouts, increased rates of delirium tremens, no sociopathy, having compulsive personality traits, being able to maintain and tolerate dependent or treatment relationships, failing with unstructured programmes, facing divorce or dismissal, being in debt or detention, losing one's house, as well as specifically requesting disulfiram. However, the evidence for these putatively good prognostic indicators is not unequivocally established. The authors also concluded that oral disulfiram seems to have some efficacy in reducing the number of drinking days and the amounts drunk in patients who are compliant with treatment regimes, even if they continued to drink. The evidence for disulfiram increasing the proportion of patients who maintain total abstinence is surprisingly lacking. In certain individuals supervised disulfiram in a comprehensive treatment programme seems to have some efficacy. Although, Garbutt *et al*[2] included only 5 studies, out of which three were double blind, they also concluded that disulfiram provides modest evidence of reduced drinking frequencies without significantly enhancing the abstinence rates and supervision significantly improved outcomes in drinking frequency and amount consumed.

We feel that disulfiram still retains its therapeutic importance in relapse prevention of alcohol.[3] It is by far the cheapest pharmacoprophylactic drug for alcohol dependence in India. To have a better understanding of efficacy of disulfiram randomized controlled trials are needed to determine whether supervised disulfiram is useful in achieving abstinence in certain high-risk groups. Cost-effectiveness studies are required to demonstrate if naltrexone and acamprosate are so effective as to clearly offset the hugely elevated cost.

## NALTREXONE

Compared to disulfiram, more number of RCT's are available for naltrexone [Table 1];[4‐17] researchers have paired naltrexone and placebo with different psychosocial therapies compare the combined efficacy. In most of the studies 50 mg/day dosing schedule has been used. Unfortunately, there is no standard set of efficacy outcome measures used in all studies. The primary outcome measure includes relapse and abstinence rates. Relapse has been defined variously as 5 or more drinks/day for men and 4 or more drinks/day for women;[5,8‐11,16] 6 or more drinks/day for men and 4 or more drinks/day for women;[15] 6 or more drinks/day for men and 5 or more drinks/day for women;[13] 5 or more days per week 5 or more drinks/episode of intake or blood alcohol concentration > 100 mg/dl.[4,6,7,12,14] In all studies abstainers were defined as those who consumed no alcohol. Secondary outcome measures include percentage of drinking days, percentage of days of abstinence, number of drinks per drinking day, days of heavy drinking and total alcohol consumption and craving. The primary outcome after administration of naltrexone is shown in Table 1. Outcome values are denoted in terms of the statistical significance of data comparing naltrexone with placebo. Thus, on each particular measure, the effects of naltrexone were either comparable to placebo (NS or nonsignificant), of significant advantage (+), or very significantly beneficial (++). In no case was naltrexone of less benefit than placebo.

**Table 1:** Double blind, placebo-controlled trials of naltrexone for the treatment of alcohol dependence

Study	No. of subjects	Therapy	Duration (weeks)	Abstinence	TTFD	Drinking Days	Drinks/drinking day	Relapse	Days of heavy drinking	Craving
Volpicelli *et al*,[4] 1995	99	Intensive multimodal	12	NS		+		+		++
O'Malley *et al*,[5] 1992	97	Coping skills or supportive	12	NS	++	+	++		+	
Volpicelli *et al*,[6] 1997	97	Relapse prevention	12	NS		+		+		NS
Oslin *et al*,[7] 1997	44	Supportive therapy	12	NS	NS	+			NS	NS
Anton *et al*,[8] 1999	131	CBT	12	NS	NS	+	++	+		NS
Hersh *et al*,[9] 1998	64	Relapse prevention	8	NS		NS	NS	NS		
Chick *et al*,[10] 2000	175	Psychosocial support	12	NS	NS	NS	+			++
Kranzler*et al*,[11] 2000	124	Behavior psychosocial	12	NS	NS	NS	NS	NS		
Morris *et al*,[12] 2000	111	Education supportive	12	NS		NS	++	++		
Monti *et al*,[13] 2001	128	Cue exposure coping skill	36	NS			+	NS	+	+
Heinala *et al*,[14] 2001	121	Coping skill, supportive	12	NS	NS			++		
Krystal *et al*,[15] 2001	12	step counseling	24			NS	NS	NS		
Guardia *et al*,[16] 2002	202	Supportive therapy	12			NS	NS	+		
Latt *et al*,[17] 2002	107	Psychosocial Support	12					+		

(+) = Significant difference in favor of medication group (*P* < 0.05)

(++)= Significant difference in favor of medication group (*P* < 0.01)

NS = No significant difference; Blank column indicates that the outcome was not reported in the study

TTFD = Time taken for first drink.

It is evident that naltrexone does not appear to exert an influence compared with placebo on maintaining abstinence or in postponing the first drink in those patients who cannot avoid alcohol. However, there is clear and consistent evidence that naltrexone is significantly beneficial in helping those patients who cannot remain abstinent to reduce their drinking behaviors. They drink less often and in lower quantities, avoiding full-blown relapse. As far as the effect on craving is concerned it is difficult to reach to a conclusion because some studies have not included it in the outcome measures, some have failed to analyze it and rest have used different instruments to measure it. Some researchers have noted that patients with higher initial craving appear to derive greatest benefit from naltrexone.[5,18‐19] Volipicelli[20] observed that naltrexone seems to have an immediate effect of reducing the urge to drink and this can be very useful in helping patients focus on other issues besides alcohol craving, especially during early stages of recovery. There is the question of why this reduced craving effect did not enhance abstinence in the RCTs. First, craving may be but one drive motivating drinking. Second, heavy drinking may itself induce craving and, since naltrexone-group patients drank less often and in lower quantities, this helps explain their lower craving scores but only equivalent abstinence compared with placebo-treated subjects.[21]

Naltrexone appears to be especially effective for patients who stay in treatment and comply with medication regimens.[5,6,13,21,22] Six of 14 RCTs examined long-term effects of naltrexone during followup periods ranging from 14 to 40 weeks after the end of drug treatment (i.e., off treatment). Results suggest that naltrexone is effective as long as it is taken, but benefits begin fading once the medication is terminated.[5,8,13–15,23,24] Some researchers have recommended a minimum of six months treatment with naltrexone.[20] It also has been proposed that, following a course of daily treatment, naltrexone can be useful on an as-needed or short-term basis; using the drug during high-risk periods or after a resumption of drinking following successful abstinence.[20,24] In their RCT, Heinala *et al*[4] included a 20 week targeted naltrexone period following daily dosing. Subjects were instructed to take naltrexone only when craving alcohol and/or drinking was likely. This intervention was of significant benefit in warding off relapse. Others have reported using this targeted-naltrexone approach effectively in reducing all measures of alcohol consumption.[11] Overall, RCTs to date have demonstrated that the incidence of subjects reporting side effects or discontinuing from naltrexone treatment due to such effects was roughly equivalent to placebo.[25] Naltrexone has been associated with increased nausea and vomiting. Less common side effects include headache, dizziness, fatigue, or insomnia. These effects are usually mild, often single occurrences and resolve soon after dose stabilization.[5,26–28] In some studies dose more than 50 mg/day has been used. Volpicelli[20] pointed out that doses of 100 mg/day up to 150 mg/day can be safely and effectively used in many patients. Monterosso *et al*[19] administered 100 mg/day (50 mg BID) in their RCT and retention/compliance rates were well above average.

Carmen *et al*[29] carried out a metanalysis of the above studies and reported that naltrexone is associated with significant improvement in relapse rate during active treatment phase, with number needed to treat (NNT) of 9 (95% CI: 6–14), as well as during follow up phase. Favorable effect is also found on abstinence rate during active treatment phase but did not reach statistical significant level, but there were no favorable effects on abstinence rate during follow up phase. There was significant benefit in the secondary outcome measures like percentage of drinking days, days of abstinence, number of drinks per drinking day, days of heavy drinking and total alcohol consumption, GGT and AST levels and craving during treatment phase. There are frequent side effects, but are not associated with any severe events.

As compliance is always an issue with the medications, authors have examined the use of long acting formulations of naltrexone. Garbutt *et al*[30] examined the safety and efficacy of an intramuscular, long-acting formulation of naltrexone in an industry-sponsored, multicentric 6-month, randomized, double-blind, placebo-controlled study conducted in the USA. The sample comprised 624 treatment-seeking alcohol-dependent adults who were randomized to receive either high dose depot naltrexone (380 mg; n = 205), low dose depot naltrexone (190 mg; n = 210), or placebo (n = 209). Treatments were administered once a month for 6 months. All patients were also exposed to 12 sessions of low-intensity psychosocial intervention.

The authors found that high dose naltrexone decreased heavy drinking days by 25% and low-dose naltrexone by 17%. The advantage for the high dose over placebo was statistically significant (*P* = 0.03) whereas that for the low dose just escaped significance (*P* = 0.07). The benefits were greater in men and in those who were abstinent before the initiation of treatment. The adverse effects of naltrexone were dose-dependent and matched the known profile of the drug. Discontinuation due to adverse events occurred in 14% of the high dose group, in 7% of the low dose group and in 7% of the placebo group. The authors concluded that depot naltrexone (380 mg i.m. once a month) is well tolerated and reduces the frequency of heavy drinking days in treatment-seeking alcohol-dependent patients studied across a 6-month period.

From the above it can be concluded that RCTs of naltrexone in the treatment of alcoholism are extensive and of good quality. There is good evidence that naltrexone significantly reduces alcohol relapses, the frequency and quantity of alcohol consumption in those who do drink and alcohol craving. In brief, naltrexone appears to break the vicious, self-destructive cycle in alcoholics whereby one drink always leads to another. On the other hand results from various studies require careful consideration. Statistical significance of outcomes is important but can be misleading, for even small improvements can be clinically and socially significant when each percentage point may represent thousands of lives benefited.

## ACAMPROSATE

Acamprosate has also been evaluated for its efficacy in substantial number of studies [Table 2][31–42] and the outcome measures studied are mostly homogenous. The outcome measures which have been evaluated include abstinence rate, cumulative abstinence duration, rate of compliance with treatment, degree of craving and hepatic enzyme levels. The abstinence rate is defined as percentage of patients that completed the study without ingesting alcohol; cumulative abstinence duration is defined as sum of the periods of abstinence during study. Similar to naltrexone, researchers have paired acamprosate and placebo with different psychosocial therapies and have compared the combined efficacy. Most of these studies have been conducted in the ambulatory setting and have used fixed doses.

**Table 2:** Placebo-controlled trials of acamprosate for the treatment of alcohol dependence

Study	No. of subjects	Therapy	Duration (weeks)	Results			
				
				CAD	Abstinence rates	Craving	Other measures
Lhuintre *et al*,[31] 1990	569	Psychotherapy	12	NR	+	NR	GOT
Ladewig, *et al*,[32] 1993	61	—	24	NR	+	NR	
Paille *et al*,[33] 1995	538	Supportive psychotherapy	52	+	+	0/+	
Sass *et al*, [34] 1996	272	—	48	+	+	NR	TTFD
Whitworth *et al*,[35] 1996	448	—	52	+	+	NR	—
Geerlings *et al*,[36] 1997	262	Psychosocial support	24	+	+	NR	
Pelc *et al*,[37] 1997	188	Counseling social support	12	+	+	+	
Poldrugo,[38] 1997	246	Rehabilitation	24	+	+	0	
Besson,[39] 1998	156	Psychosocial support	52	+	+	+	
Chick *et al*,[40] 2000	581	Marital, social skill training	4	NR	+	+	
Tempesta *et al*,[41] 2000	330	Behavior oriented supportive	12	+	0/+	0/+	TTFD
Gual and Lehert,[42] 2001	288	Psychosocial support	24	+	+	+	

(+) = Significant difference in favor of medication group

(-) = Significant difference in favor of placebo group

TTFD = Time to first drink

NR = Outcome was not reported

Most of the trials have reported significantly favourable effect of acamprosate on abstinence rate, CAD and rate of compliance with treatment. Results regarding the effect on craving vary. Chick *et al*[40] observed a significantly favorable effect for acamprosate on assessment at 1 month, other authors failed to observe significant differences versus the control group at 12 months of treatment, whether in terms of percentage of patients without craving,[42] change over base line[43] or mean value on visual analogue scale.[34] With respect to GGT, 11 studies included this as an outcome measure and reported significantly favourable trend in the acamprosate group. Acamprosate produced few side effects with mainly diarrhoea and occasionally, headache, dizziness and Pruritus.

Six meta-analyses of the efficacy of acamprosate in the treatment of alcohol dependence have been undertaken to date, all of which have concluded that acamprosate is effective in maintaining abstinence in detoxified alcohol dependent individuals.[29,44–48] Out of the six, only 3 have been published in a peer-reviewed journal.[29,45,48]

Kranzler and Van Kirk[45] carried out metanalysis of available studies for both acamprosate and naltrexone and concluded that both the medications significantly affects drinking outcomes. The most robust effect of these medications appeared to be on the frequency of drinking, as reflected by either percentage drinking days (in the naltrexone studies) or cumulative abstinent days (in the acamprosate studies). However, significant heterogeneity among studies was found for both of these measures. Based on limited comparisons of the two medications, there appeared to be no statistical difference in their efficacy in the treatment of alcohol dependence. Mann *et al*[48] found that acamprosate has a significant beneficial effect in enhancing abstinence in recently detoxified, alcohol-dependent individuals. Continuous abstinence rates at 6 months were significantly higher in the acamprosate-treated patients. This effect was observed independently of the method used for assigning missing data. The effect sizes in abstinent rates at 3, 6 and 12 months were 1.33, 1.50 and 1.95, respectively. At 12 months, the overall pooled difference in success rates between acamprosate and placebo was 13.3% (95% CI, 7.8–18.7%; number needed to treat, 7.5). Acamprosate also had a modest but significant beneficial effect on retention (6.01%; [95% CI, 2.90–8.82]; *P* = 0.0106).

Carmen *et al*[29] carried out a metanalysis of 12 studies and concluded that acamprosate raised the continuous abstinence rate with a calculate NNT of 10 (95% CI: 7–15). Treatment with acamprosate was associated with significantly favorable effects in cumulative abstinence duration. In fact, acamprosate doubled the days of cumulative abstinence in the seven studies that supplied the data. Because of the diversity in the expression in the expression of secondary outcomes, the effects couldn't be estimated. Regarding the GGT levels, because of disparity and inadequate description of results and even lack of quantitative data, the pooled effect estimation could not be done. The metanalysis from 10 studies showed that gastrointestinal adverse effects were significantly more common in the acamprosate groups than in the control group. Nevertheless, no statistically significant differences were observed between groups in terms of premature withdrawals from treatment due to adverse effects. Acamprosate improved overall adherence to treatment with a calculated NNT of 16 (95 5 CI: 11–33).

From the above it can be concluded that acamprosate is a safe therapy that prolongs abstinence and reduces the rate of relapse among alcohol dependent subjects. The effect on craving is still controversial and needs evaluation and with the current level of evidence it would be appropriate to call acamprosate as a “relapse preventing agent” rather than an “anticraving agent.” In addition it also appears to be safe in patients with hepatic dysfunction, which is a major concern with other two drugs.

## COMPARISON OF TWO DRUGS

As discussed earlier, there are only 3 trials that have compared the above drugs with each other, out of which two are from India and one is from our centre. In two of these, acamprosate has been compared with naltrexone and in another study naltrexone was compared with disulfiram. In the comparison study by Rubio *et al*[49] naltrexone led to significantly more abstinence rate and relapse, longer time to relapse, more reduction in number of drinks per drinking day, better reduction of craving and better retention rates compared to acamprosate. But the adverse effects, both gastrointestinal and neuropsychiatric were higher in naltrexone group.

In the study by Naidu *et al*,[50] in a 24 weeks trial found subjects on naltrexone had significantly less craving for alcohol, reported significantly less side effects and higher decline in GGT levels at the end of the study compared to disulfiram. Fifty percent of the naltrexone group relapsed compared to 77.7% in the disulfiram group.

In a recent retrospective chart review from our centre, Basu *et al*[51] compared those on acamprosate or naltrexone vis-à-vis those on no prophylactic drugs with respect to their demographic and clinical background and short-term outcome after treatment. Compared to those on naltrexone or no drugs, significantly more patients on acamprosate came from higher socioeconomic strata, had less family/marital complications and less comorbid use of opioids and other drugs; however, they also had more liver function impairment, alcoholic liver disease and higher average duration of relapse in the past (*P* < 0.05 or less in each case). The group on no drugs had significantly less family/social support (*P* = 0.006) and poorer motivation rating (*P* < 0.001) than the other two groups on drugs. Intent-to-treat analysis showed that there was a non-significant trend of a higher proportion of acamprosate patients remaining abstinent (77%) than those on naltrexone (36%) and no drug (50%). At follow-up, acamprosate patients had significantly better functioning in several areas. However, because many of the baseline patient characteristics might themselves have influenced the outcome, hence no conclusion should be drawn from this data regarding the efficacy of the drug. Logistic regression analysis showed that both family/social support and acamprosate appeared to contribute modestly towards explaining the variance in short-term outcome.

## COMBINATION OF DRUGS

In a study (n = 160), Kiefer *et al*[52] found that a combination of naltrexone (50 mg/day) and acamprosate (666 mg thrice a day) yielded the best results on multiple outcome measures in young, predominantly male, detoxified chronic alcoholics. They randomized the patients to receive naltrexone, acamprosate, naltrexone plus acamprosate, or placebo for 12 weeks. Patients were assessed weekly by interview, self-report, questionnaires and laboratory screening. Time to first drink, time to relapse and the cumulative abstinence time were the primary outcome measures. They found that naltrexone, acamprosate and the combined medication were significantly more effective than placebo. Comparing the course of non-relapse rates between naltrexone and acamprosate, the naltrexone group showed a tendency for a better outcome regarding time to first drink and time to relapse. The combined medication was most effective with significantly lower relapse rates than placebo and acamprosate but not naltrexone. They concluded that naltrexone and acamprosate, especially in combination, considerably enhance the potential of relapse prevention.

Treatment of alcohol disorders through the use of combinations of pharmacological and behavioral modalities may more effectively address the multicomponent nature of the disorder than single-modality approaches. Interdisciplinary models of the biological, psychological and social components of alcohol disorders are emerging rapidly from basic research and treatment researchers have begun to test various strategies to combine medications and behavioral treatments. In addition to behavioral and pharmacological combinations, effective treatment pairs can be composed of two medications whose mechanisms of action are believed to be compatible and potentially additive, or even synergistic. Combining Medications and Behavioral Interventions (COMBINE) is a large multisite clinical trial sponsored by the National Institute on Alcohol Abuse and Alcoholism.[53] Its goal is to determine if improvements in treatment outcome for alcohol dependence can be achieved by combining pharmacotherapy and behavioral interventions. Under evaluation is the efficacy of two promising medications (naltrexone and acamprosate), both singly and together, when used in conjunction with two behavioral treatments of differing intensities. The results are awaited.[54]

## TOPIRAMATE

Topiramate is a newer drug in this class and there are only three studies that have evaluated its efficacy in alcohol dependent subjects. Johnson *et al*[55] conducted a randomized, double-blind, placebo-controlled clinical trial for 12 weeks using topiramate in dosages of up to 300 mg daily. The 150 participants, who were 21 to 65 years of age, met criteria in diagnostic and statistical manual of mental disorders, 4^th^ ed. (DSM-IV) for alcohol dependence, had diagnostic scores on tests of alcohol-use disorders and reported drinking at least 21 (for participating women) or 35 (for participating men) standard drinks per week in the 90 days before the study. To be included in the study, participants had to have urine toxicology screens that were negative for narcotics, amphetamines, or sedative hypnotics. After a comprehensive intake assessment, participants were randomly assigned to treatment with topiramate or placebo. The starting dosage of topiramate, 25 mg daily, was escalated over eight weeks to 300 mg daily. If tolerated, this dosage was maintained for the final four weeks of the study. All patients received brief behavior compliance-enhancement treatment weekly from nurse practitioners who dispensed medications and adherence was checked weekly by physicians. Patient compliance with the study was assessed by breath alcohol measurements and other indicators of drinking at the weekly visits, plus counts of returned pill packs. The primary outcome of self-reported drinking was assessed by the number of drinks per day, the percentage of heavy drinking days and the number of days of abstinence. Craving was assessed using a standardized 14-item scale. Objective evidence of drinking was obtained by checking the plasma gamma-glutamyl transferase concentration. By the end of the study, patients receiving topiramate were significantly more successful than those taking placebo in reducing the number of drinks per day, the number of drinks on each drinking day, the percentage of heavy drinking days and measures of gamma-glutamyl transferase. They also significantly increased the percentage of abstinent days compared with the placebo group. The significant differences between the groups became apparent at weeks 6 and 8 and increased as the study progressed. Compared with the placebo group, by the end of the study the patients treated with topiramate reported having 2.88 fewer drinks per day, 3.10 fewer drinks per drinking day, 27.6 fewer days of heavy drinking and 26.2 more days of abstinence. Changes in the gamma-glutamyl transferase ratio indicated significantly decreased alcohol intake in the treated group. These differences between the groups also were apparent in measures of cravings during treatment. Dizziness, parasthesias, psychomotor slowing, memory impairment and weight loss were reported more frequently by patients taking topiramate, but only three patients withdrew from the topiramate group and five from the placebo group because of adverse effects. The authors concluded that topiramate is more effective than placebo as an adjunct treatment of alcohol dependence in a 12-week program. The effect appears to become significant at dosages of 200 mg daily but increases over time as the dosage is increased to a maximum of 300 mg daily; therefore, independent effects of time and dosage cannot be segregated.

In another study, of 12 weeks duration, open labeled in design, Rubio *et al*[56] studied 24 patients who fulfilled alcohol-dependence criteria as per DSM-IV. Topiramate was used as adjunctive therapy at an initial dose of 50 mg/day and titrated upwards by 25 mg every 3 days up to a maximum dose of 400 mg/day. Topiramate was well tolerated and only 3 subjects dropped out due to adverse events. All the subjects who completed the trial had improvement in visual-analogic scale for craving severity, weekly drink consumption and carbohydrate-deficient transferring levels.

In another important recent study, Johnson *et al*[57] studied whether topiramate, compared with placebo, improves psychosocial functioning in alcohol-dependent individuals and how this improvement is related to heavy drinking behavior. In a double-blind, randomized, controlled, 12-week clinical trial comparing topiramate vs. placebo for treating alcohol dependence, 150 alcohol-dependent individuals, diagnosed using the DSM-IV, received either topiramate (escalating dose of 25 mg/d to 300 mg/d) or placebo plus weekly standardized medication compliance management. Three elements of psychosocial functioning were measured: clinical ratings of overall well-being and alcohol-dependence severity, quality of life and harmful drinking consequences. The results showed that, topiramate, compared with placebo, improved the odds of overall well-being (odds ratio [OR] = 2.17; 95% confidence interval [CI], 1.16-2.60; *P* = 0.01); reported abstinence and not seeking alcohol (OR = 2.63; 95% CI, 1.52–4.53; *P* = 0.001); overall life satisfaction (OR = 2.28; 95% CI, 1.21–4.29; *P* = 0.01); and reduced harmful drinking consequences (OR = −0.07; 95% CI, −0.12 to −0.02, *P* = 0.01). There was a significant shift from higher to lower drinking quartiles on percentage of heavy drinking days, which was associated with improvements on all measures of psychosocial functioning. The authors concluded that, as an adjunct to medication compliance enhancement treatment, topiramate (up to 300 mg/d) was superior to placebo at not only improving drinking outcomes but also increasing overall well-being and quality of life and lessening dependence severity and its harmful consequences.

## INTRAMUSCULAR DEPOT NALTREXONE

Although oral naltrexone has been shown to diminish alcohol reinforcement, its limitations as a medication include its small treatment effect size, plasma level fluctuation and adverse events. The pharmacokinetic profile of naltrexone could be optimised by intramuscular administration, sustaining its release over several weeks. As a result, plasma levels would remain relatively constant; high enough to reduce drinking, low enough to minimise side effects. Two injectable naltrexone depot preparations, Vivitrex^R^ and Naltrel^R^, have been tested as pharmacotherapy for alcohol dependence. Their adverse-event profiles seem to be mild compared with oral naltrexone.[58]

Other than the above issues, as compliance is always an issue with the oral medications, researchers have examined the use of long acting formulations of naltrexone. Garbutt *et al*[30] examined the safety and efficacy of an intramuscular, long-acting formulation of naltrexone in an industry-sponsored, multicentric 6-month, randomized, double-blind, placebo-controlled study conducted in the USA. The sample comprised 624 treatment-seeking alcohol-dependent adults who were randomized to receive either high dose depot naltrexone (380 mg; n = 205), low-dose depot naltrexone (190 mg; n = 210), or placebo (n = 209). Treatments were administered once a month for 6 months. All patients were also exposed to 12 sessions of low-intensity psychosocial intervention. The authors found that high dose naltrexone decreased heavy drinking days by 25% and low-dose naltrexone by 17%. The advantage for the high dose over placebo was statistically significant (*P* = 0.03) whereas that for the low dose just escaped significance (*P* = 0.07). The benefits were greater in men and in those who were abstinent before the initiation of treatment. The adverse effects of naltrexone were dose-dependent and matched the known profile of the drug. Discontinuation due to adverse events occurred in 14% of the high dose group, in 7% of the low dose group and in 7% of the placebo group. The authors concluded that depot naltrexone (380 mg i.m. once a month) is well tolerated and reduces the frequency of heavy drinking days in treatment-seeking alcohol-dependent patients studied across a 6-month period.

It seems that with more efficacy studies published, injectable depot preparations of naltrexone can overcome many of the limitations of oral therapy and thus hold the potential of ushering in a new era of promise in this important area.[58]

## CONCLUSION

Alcohol dependence is a major problem in India. The pharmacological armamentarium for relapse prevention of alcohol has widened with the addition of new drugs. In this article, we review the pharmacology and efficacy of the four most important such drugs: disulfiram, naltrexone, acamprosate and topiramate. Overall, all four of these drugs have modest but clinically significant usefulness as pharmacoprophylactic agents for relapse prevention or minimization of alcohol dependence. Combinations might be helpful, especially for naltrexone and acamprosate. The issue of supervision and compliance remains important, especially for such drugs as disulfiram and naltrexone. Topiramate is a promising new agent and requires further study. Disulfiram, while very effective in compliant patients, presents challenges in terms of patient selection and side effects. For patients with hepatic impairment, acamprosate is a good choice. Finally, the latest advent of depot-preparation naltrexone may herald a new era in this important area.
